# Inferring Expected Runtimes of Probabilistic Integer Programs Using Expected Sizes

**DOI:** 10.1007/978-3-030-72016-2_14

**Published:** 2021-03-01

**Authors:** Fabian Meyer, Marcel Hark, Jürgen Giesl

**Affiliations:** 8 Eindhoven University of Technology, Eindhoven, The Netherlands; 9 Aalborg University, Aalborg East, Denmark; LuFG Informatik 2, RWTH Aachen University, Aachen, Germany

## Abstract

We present a novel modular approach to infer upper bounds on the expected runtimes of probabilistic integer programs automatically. To this end, it computes bounds on the runtimes of program parts and on the sizes of their variables in an alternating way. To evaluate its power, we implemented our approach in a new version of our open-source tool KoAT.

## 1 Introduction

There exist several approaches and tools for automatic complexity analysis of non-probabilistic programs, e.g., [2–6, 8, 9, 18, 20, 21, 27, 28, 30, 34–36, 51, 57, 58]. While most of them rely on basic techniques like *ranking functions* (see, e.g., [6, 12–14, 17, 53]), they usually combine these basic techniques in sophisticated ways. For example, in [18] we developed a modular approach for automated complexity analysis of integer programs, based on an alternation between finding symbolic runtime bounds for program parts and using them to infer bounds on the sizes of variables in such parts. So each analysis step is restricted to a small part of the program. The implementation of this approach in KoAT [18] (which is integrated in AProVE [30]) is one of the leading tools for complexity analysis [31].

While there exist several adaptions of basic techniques like ranking functions to *probabilistic programs* (e.g., [1, 11, 15, 16, 22–26, 29, 32, 37, 38, 48, 62]), most of the sophisticated full approaches for complexity analysis have not been adapted to probabilistic programs yet, and there are only few powerful tools available which analyze the runtimes of probabilistic programs automatically [10, 50, 61, 62].

We study probabilistic integer programs (Sect. 2) and define suitable notions of non-probabilistic and expected runtime and size bounds (Sect. 3). Then, we adapt our modular approach for runtime and size analysis of [18] to probabilistic programs (Sect. 4 and 5). So such an adaption is not only possible for *basic techniques* like ranking functions, but also for *full approaches* for complexity analysis.

For this adaption, several problems had to be solved. When computing expected runtime or size bounds for new program parts, the main difficulty is to determine when it is sound to use *expected* bounds on previous program parts and when one has to use *non-probabilistic* bounds instead. Moreover, the semantics of probabilistic programs is significantly different from classical integer programs. Thus, the proofs of our techniques differ substantially from the ones in [18], e.g.,

* funded by the Deutsche Forschungsgemeinschaft (DFG, German Research Foundation) - 235950644 (Project GI 274/6-2) & DFG Research Training Group 2236 UnRAVeL

we have to use concepts from measure theory like ranking supermartingales.

In Sect. 6, we evaluate the implementation of our new approach in the tool KoAT [18,43] and compare with related work. We refer to [47] for an appendix of our paper containing all proofs, preliminaries from probability and measure theory, and an overview on the benchmark collection used in our evaluation.

## 2 Probabilistic Integer Programs

For any set $M \subseteq \overline{\mathbb{R}}$ (with $\overline{\mathbb{R}} = \mathbb{R} \cup \{\infty\}$) and $w \in M$, let $M_{\geq w} = \{v \in M \mid v \geq w \vee v = \infty\}$. For a set $\mathcal{PV}$ of *program variables*, we first introduce the kind of *bounds* that our approach computes. Similar to [18], our bounds represent *weakly monotonically increasing* functions from $\mathcal{PV} \to \overline{\mathbb{R}}_{\geq 0}$. Such bounds have the advantage that they can easily be "composed", i.e., if $f$ and $g$ are both weakly monotonically increasing upper bounds, then so is $f \circ g$.

**Definition 1 (Bounds).** *The set of bounds $\mathcal{B}$ is the smallest set with $\mathcal{PV} \cup \overline{\mathbb{R}}_{\geq 0} \subseteq \mathcal{B}$, and where $b_1, b_2 \in \mathcal{B}$ and $v \in \mathbb{R}_{\geq 1}$ imply $b_1 + b_2,\ b_1 \cdot b_2 \in \mathcal{B}$ and $v^{b_1} \in \mathcal{B}$.*

Our notion of probabilistic programs combines classical integer programs (as in, e.g., [18]) and probabilistic control flow graphs (see, e.g., [1]). A *state* $s$ is a variable assignment $s\colon \mathcal{V} \to \mathbb{Z}$ for the (finite) set $\mathcal{V}$ of all variables in the program, where $\mathcal{PV} \subseteq \mathcal{V}$, $\mathcal{V} \setminus \mathcal{PV}$ is the set of *temporary variables*, and $\Sigma$ is the set of all states. For any $s \in \Sigma$, the state $|s|$ is defined by $|s|(x) = |s(x)|$ for all $x \in \mathcal{V}$. The set $\mathcal{C}$ of *constraints* is the smallest set containing $e_1 \leq e_2$ for all polynomials $e_1, e_2 \in \mathbb{Z}[\mathcal{V}]$ and $c_1 \wedge c_2$ for all $c_1, c_2 \in \mathcal{C}$. In addition to "$\leq$", in examples we also use relations like "$>$", which can be simulated by constraints (e.g., $e_1 > e_2$ is equivalent to $e_2 + 1 \leq e_1$ when regarding integers). We also allow the application of states to arithmetic expressions $e$ and constraints $c$. Then the number $s(e)$ resp. $s(c) \in \{\mathbf{t}, \mathbf{f}\}$ results from evaluating the expression resp. the constraint when substituting every variable $x$ by $s(x)$. So for bounds $b \in \mathcal{B}$, we have $|s|(b) \in \overline{\mathbb{R}}_{\geq 0}$.

In the transitions of a program, a program variable $x \in \mathcal{PV}$ can also be updated by adding a value according to a *bounded distribution function* $d : \Sigma \to \text{Dist}(\mathbb{Z})$. Here, for any state $s$, $d(s)$ is the probability distribution of the values that are added to $x$. As usual, a *probability distribution* on $\mathbb{Z}$ is a mapping $pr : \mathbb{Z} \to \mathbb{R}$ with $pr(v) \in [0, 1]$ for all $v \in \mathbb{Z}$ and $\sum_{v \in \mathbb{Z}} pr(v) = 1$. Let $\text{Dist}(\mathbb{Z})$ be the set of distributions $pr$ whose expected value $\mathbb{E}(pr) = \sum_{v \in \mathbb{Z}} v \cdot pr(v)$ is well defined and finite, i.e., $\mathbb{E}_{\text{abs}}(pr) = \sum_{v \in \mathbb{Z}} |v| \cdot pr(v) < \infty$. A distribution function $d : \Sigma \to \text{Dist}(\mathbb{Z})$ is *bounded* if there is a finite bound $\mathfrak{E}(d) \in \mathcal{B}$ with $\mathbb{E}_{\text{abs}}(d(s)) \leq |s|(\mathfrak{E}(d))$ for all $s \in \Sigma$. Let $\mathcal{D}$ denote the set of all bounded distribution functions (our implementation supports Bernoulli, uniform, geometric, hypergeometric, and binomial distributions, see [43] for details).

**Definition 2 (PIP).** *$(\mathcal{PV}, \mathcal{L}, \mathcal{GT}, \ell_0)$ is a* probabilistic integer program *with*

1. *a finite set of* program variables $\mathcal{PV} \subseteq \mathcal{V}$
2. *a finite non-empty set of program* locations $\mathcal{L}$
3. *a finite non-empty set of* general transitions $\mathcal{GT}$. *A general transition $g$ is a finite non-empty set of transitions $t = (\ell, p, \tau, \eta, \ell')$, consisting of*

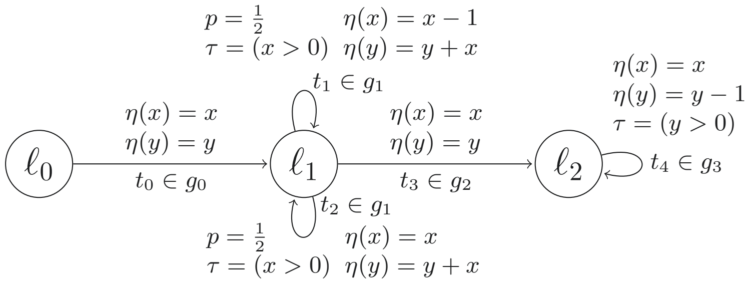

Fig. 1: PIP with non-deterministic and probabilistic branching

*(a) the* start *and* target locations $\ell, \ell' \in \mathcal{L}$ *of transition* $t$*,*
*(b) the* probability $p \geq 0$ *that transition* $t$ *is chosen when* $g$ *is executed,*
*(c) the* guard $\tau \in \mathcal{C}$ *of* $t$*, and*
*(d) the* update function $\eta\colon \mathcal{PV} \to \mathbb{Z}[\mathcal{V}] \cup \mathcal{D}$ *of* $t$*, mapping every program variable to an update polynomial or a bounded distribution function.*

*All* $t \in g$ *must have the same start location* $\ell$ *and the same guard* $\tau$*. Thus, we call them the start location and guard* of $g$*, and denote them by* $\ell_g$ *and* $\tau_g$*. Moreover, the probabilities* $p$ *of the transitions in* $g$ *must add up to* 1*.*

*4. an* initial location $\ell_0 \in \mathcal{L}$*, where no transition has target location* $\ell_0$

PIPs allow for both probabilistic and non-deterministic branching and sampling. Probabilistic branching is modeled by selecting a transition out of a non-singleton general transition. Non-deterministic branching is represented by several general transitions with the same start location and non-exclusive guards. Probabilistic sampling is realized by update functions that map a program variable to a bounded distribution function. Non-deterministic sampling is modeled by updating a program variable with an expression containing temporary variables from $\mathcal{V} \setminus \mathcal{PV}$, whose values are non-deterministic (but can be restricted in the guard). The set of *initial* general transitions $\mathcal{GT}_0 \subseteq \mathcal{GT}$ consists of all general transitions with start location $\ell_0$.

*Example 3 (PIP). Consider the PIP in Fig. 1 with initial location* $\ell_0$ *and the program variables* $\mathcal{PV} = \{x, y\}$*. Here, let* $p = 1$ *and* $\tau = \mathbf{t}$ *if not stated explicitly. There are four general transitions:* $g_0 = \{t_0\}$*,* $g_1 = \{t_1, t_2\}$*,* $g_2 = \{t_3\}$*, and* $g_3 = \{t_4\}$*, where* $g_1$ *and* $g_2$ *represent a non-deterministic branching. When choosing the general transition* $g_1$*, the transitions* $t_1$ *and* $t_2$ *encode a probabilistic branching. If we modified the update* $\eta$ *and the guard* $\tau$ *of* $t_0$ *to* $\eta(x) = u \in \mathcal{V} \setminus \mathcal{PV}$ *and* $\tau = (u > 0)$*, then* $x$ *would be updated to a non-deterministically chosen positive value. In contrast, if* $\eta(x) = \mathrm{GEO}(\frac{1}{2})$*, then* $t_0$ *would update* $x$ *by adding a value sampled from the geometric distribution with parameter* $\frac{1}{2}$*.*

In the following, we regard a fixed PIP $\mathcal{P}$ as in Def. 2. A *configuration* is a tuple $(\ell, t, s)$, with the current location $\ell \in \mathcal{L}$, the current state $s \in \Sigma$, and the transition $t$ that was evaluated last and led to the current configuration. Let $\mathcal{T} = \bigcup_{g \in \mathcal{GT}} g$. Then $\mathsf{Conf} = (\mathcal{L} \uplus \{\ell_\bot\}) \times (\mathcal{T} \uplus \{t_{\mathrm{in}}, t_\bot\}) \times \Sigma$ is the set of all configurations, with a special location $\ell_\bot$ indicating the termination of a run, and special transitions $t_{\mathrm{in}}$ (used in the first configuration of a run) and $t_\bot$ (for the configurations of the run after termination). The (virtual) general transition $g_\perp = \{t_\perp\}$ only contains $t_\perp$.

A *run* of a PIP is an infinite sequence $\vartheta = c_0\, c_1 \cdots \in \mathsf{Conf}^\omega$. Let $\mathsf{Runs} = \mathsf{Conf}^\omega$ and let $\mathsf{FPath} = \mathsf{Conf}^*$ be the set of all *finite paths* of configurations.

In our setting, deterministic Markovian schedulers suffice to resolve all non-determinism (see, e.g., [54, Prop. 6.2.1]). For $c = (\ell, t, s) \in \mathsf{Conf}$, such a *scheduler* $\mathfrak{S}$ yields a pair $\mathfrak{S}(c) = (g, s')$ where $g$ is the next general transition to be taken (with $\ell = \ell_g$) and $s'$ chooses values for the temporary variables where $s'(\tau_g) = \mathsf{t}$ and $s(x) = s'(x)$ for all $x \in \mathcal{PV}$. If $\mathcal{GT}$ contains no such $g$, we get $\mathfrak{S}(c) = (g_\perp, s)$.

For each scheduler $\mathfrak{S}$ and initial state $s_0$, we first define a probability mass function $pr_{\mathfrak{S},s_0}$. For all $c \in \mathsf{Conf}$, $pr_{\mathfrak{S},s_0}(c)$ is the probability that a run starts in $c$. Thus, $pr_{\mathfrak{S},s_0}(c) = 1$ if $c = (\ell_0, t_{\text{in}}, s_0)$ and $pr_{\mathfrak{S},s_0}(c) = 0$, otherwise. Moreover, for all $c', c \in \mathsf{Conf}$, $pr_{\mathfrak{S},s_0}(c' \to c)$ is the probability that the configuration $c'$ is followed by the configuration $c$ (see [47] for the formal definition of $pr_{\mathfrak{S},s_0}$).

For any $f = c_0 \cdots c_n \in \mathsf{FPath}$, let $pr_{\mathfrak{S},s_0}(f) = pr_{\mathfrak{S},s_0}(c_0) \cdot pr_{\mathfrak{S},s_0}(c_0 \to c_1) \cdot \ldots \cdot pr_{\mathfrak{S},s_0}(c_{n-1} \to c_n)$. We say that $f$ is *admissible* for $\mathfrak{S}$ and $s_0$ if $pr_{\mathfrak{S},s_0}(f) > 0$. A run $\vartheta$ is admissible if all its finite prefixes are admissible. A configuration $c \in \mathsf{Conf}$ is admissible if there is some admissible finite path ending in $c$.

The semantics of PIPs can now be defined by giving a corresponding probability space, which is obtained by a standard cylinder construction (see, e.g., [7,60]). Let $\mathbb{P}_{\mathfrak{S},s_0}$ denote the corresponding probability measure which lifts $pr_{\mathfrak{S},s_0}$ to cylinder sets: For any $f \in \mathsf{FPath}$, we have $pr_{\mathfrak{S},s_0}(f) = \mathbb{P}_{\mathfrak{S},s_0}(\mathrm{Pre}_f)$ for the set $\mathrm{Pre}_f$ of all runs with prefix $f$. So $\mathbb{P}_{\mathfrak{S},s_0}(\Theta)$ is the probability that a run from $\Theta \subseteq \mathsf{Runs}$ is obtained when using the scheduler $\mathfrak{S}$ and starting in $s_0$.

We denote the associated expected value operator by $\mathbb{E}_{\mathfrak{S},s_0}$. So for any random variable $X : \mathsf{Runs} \to \overline{\mathbb{N}} = \mathbb{N} \cup \{\infty\}$, we have $\mathbb{E}_{\mathfrak{S},s_0}(X) = \sum_{n \in \overline{\mathbb{N}}} n \cdot \mathbb{P}_{\mathfrak{S},s_0}(X = n)$. For details on the preliminaries from probability theory we refer to [47].

## 3 Complexity Bounds

In Sect. 3.1, we first recapitulate the concepts of (non-probabilistic) runtime and size bounds from [18]. Then we introduce *expected* runtime and size bounds in Sect. 3.2 and connect them to their non-probabilistic counterparts.

### 3.1 Runtime and Size Bounds

Again, let $\mathcal{P}$ denote the PIP which we want to analyze. Def. 4 recapitulates the notions of runtime and size bounds from [18] in our setting. Recall that bounds from $\mathcal{B}$ do not contain temporary variables, i.e., we always try to infer bounds in terms of the initial values of the *program variables*. Let $\sup \varnothing = 0$, as all occurring sets are subsets of $\overline{\mathbb{R}}_{\geq 0}$, whose minimal element is 0.

**Definition 4 (Runtime and Size Bounds [18]).** *$\mathcal{RB}\colon \mathcal{T} \to \mathcal{B}$ is a* runtime bound *and $\mathcal{SB}\colon \mathcal{T} \times \mathcal{V} \to \mathcal{B}$ is a* size bound *if for all transitions $t \in \mathcal{T}$, all variables $x \in \mathcal{V}$, all schedulers $\mathfrak{S}$, and all states $s_0 \in \Sigma$, we have*

$$\begin{aligned} |s_0|\,(\mathcal{RB}(t)) &\geq \sup\{\, |\{i \mid t_i = t\}| \mid f = (\_, t_0, \_) \cdots (\_, t_n, \_) \wedge pr_{\mathfrak{S},s_0}(f) > 0 \,\},\\ |s_0|\,(\mathcal{SB}(t,x)) &\geq \sup\{\, |s(x)| \mid f = \cdots (\_, t, s) \wedge pr_{\mathfrak{S},s_0}(f) > 0 \,\}. \end{aligned}$$

So $\mathcal{RB}(t)$ is a bound on the number of executions of $t$ and $\mathcal{SB}(t, x)$ over-approximates the greatest absolute value that $x \in \mathcal{V}$ takes after the application of the transition $t$ in any admissible finite path. Note that Def. 4 does not apply to $t_{\text{in}}$ and $t_\perp$, since they are not contained in $\mathcal{T}$.

We call a tuple $(\mathcal{RB}, \mathcal{SB})$ a (non-probabilistic) *bound pair*. We will use such non-probabilistic bound pairs for an initialization of expected bounds (Thm. 10) and to compute improved expected runtime and size bounds in Sect. 4 and 5.

*Example 5 (Bound Pair). The technique of [18] computes the following bound pair for the PIP of Fig. 1 (by ignoring the probabilities of the transitions).*

$$\mathcal{RB}(t) = \begin{cases} 1, & \text{if } t = t_0 \text{ or } t = t_3 \\ x, & \text{if } t = t_1 \\ \infty, & \text{if } t = t_2 \text{ or } t = t_4 \end{cases} \qquad \mathcal{SB}(t, x) = \begin{cases} x, & \text{if } t \in \{t_0, t_1, t_2\} \\ 3 \cdot x, & \text{if } t \in \{t_3, t_4\} \end{cases}$$

$$\mathcal{SB}(t, y) = \begin{cases} y, & \text{if } t = t_0 \\ \infty, & \text{if } t \in \{t_1, t_2, t_3, t_4\} \end{cases}$$

*Clearly, $t_0$ and $t_3$ can only be evaluated once. Since $t_1$ decrements $x$ and no transition increments it, $t_1$'s runtime is bounded by $|s_0|(x)$. However, $t_2$ can be executed arbitrarily often if $s_0(x) > 0$. Thus, the runtimes of $t_2$ and $t_4$ are unbounded (i.e., $\mathcal{P}$ is not terminating when regarding it as a non-probabilistic program). $\mathcal{SB}(t, x)$ is finite for all transitions $t$, since $x$ is never increased. In contrast, the value of $y$ can be arbitrarily large after all transitions but $t_0$.*

### 3.2 Expected Runtime and Size Bounds

We now define the *expected* runtime and size complexity of a PIP $\mathcal{P}$.

**Definition 6 (Expected Runtime Complexity, PAST [15]).** *For $g \in \mathcal{GT}$, its* runtime *is the random variable $\mathcal{R}(g)$ where $\mathcal{R}\colon \mathcal{GT} \to \mathsf{Runs} \to \overline{\mathbb{N}}$ with*

$$\mathcal{R}(g)(\,(\_, t_0, \_)\,(\_, t_1, \_)\,\cdots\,) \;=\; |\,\{i \mid t_i \in g\}\,|.$$

*For a scheduler $\mathfrak{S}$ and $s_0 \in \Sigma$, the* expected runtime complexity *of $g \in \mathcal{GT}$ is $\mathbb{E}_{\mathfrak{S},s_0}(\mathcal{R}(g))$ and the* expected runtime complexity *of $\mathcal{P}$ is $\sum_{g \in \mathcal{GT}} \mathbb{E}_{\mathfrak{S},s_0}(\mathcal{R}(g))$.*

*If $\mathcal{P}$'s expected runtime complexity is finite for every scheduler $\mathfrak{S}$ and every initial state $s_0$, then $\mathcal{P}$ is called* positively almost surely terminating (PAST).

So $\mathcal{R}(g)(\vartheta)$ is the number of executions of a transition from $g$ in the run $\vartheta$.

While non-probabilistic size bounds refer to pairs $(t, x)$ of transitions $t \in \mathcal{T}$ and variables $x \in \mathcal{V}$ (so-called *result variables* in [18]), we now introduce expected size bounds for *general result variables* $(g, \ell, x)$, which consist of a general transition $g$, one of its target locations $\ell$, and a program variable $x \in \mathcal{PV}$. So $x$ must not be a temporary variable (which represents *non-probabilistic* non-determinism), since general result variables are used for *expected* size bounds.

**Definition 7 (Expected Size Complexity).** *The set of* general result variables *is $\mathcal{GRV} = \{\,(g, \ell, x) \mid g \in \mathcal{GT}, x \in \mathcal{PV}, (\_, \_, \_, \_, \ell) \in g\,\}$. The* size *of $\alpha = (g, \ell, x) \in \mathcal{GRV}$ is the random variable $\mathcal{S}(\alpha)$ where $\mathcal{S}\colon \mathcal{GRV} \to \mathsf{Runs} \to \overline{\mathbb{N}}$ with*

$$\mathcal{S}(g, \ell, x)\,(\,(\ell_0, t_0, s_0)\,(\ell_1, t_1, s_1)\cdots\,) \;=\; \sup\{\,|s_i(x)| \;\mid\; \ell_i = \ell \wedge t_i \in g\,\}\,.$$

*For a scheduler* $\mathfrak{S}$ *and* $s_0$*, the* expected size complexity *of* $\alpha \in \mathcal{GRV}$ *is* $\mathbb{E}_{\mathfrak{S},s_0}(\mathcal{S}(\alpha))$.

So for any run $\vartheta$, $\mathcal{S}(g,\ell,x)(\vartheta)$ is the greatest absolute value of $x$ in location $\ell$, whenever $\ell$ was entered with a transition from $g$. We now define bounds for the expected runtime and size complexity which hold *independent* of the scheduler.

**Definition 8 (Expected Runtime and Size Bounds).**

- $\mathcal{RB}_{\mathbb{E}} : \mathcal{GT} \to \mathcal{B}$ *is an* expected runtime bound *if for all* $g \in \mathcal{GT}$*, all schedulers* $\mathfrak{S}$*, and all* $s_0 \in \Sigma$*, we have* $|s_0|\,(\mathcal{RB}_{\mathbb{E}}(g)) \geq \mathbb{E}_{\mathfrak{S},s_0}(\mathcal{R}(g))$.
- $\mathcal{SB}_{\mathbb{E}} : \mathcal{GRV} \to \mathcal{B}$ *is an* expected size bound *if for all* $\alpha \in \mathcal{GRV}$*, all schedulers* $\mathfrak{S}$*, and all* $s_0 \in \Sigma$*, we have* $|s_0|\,(\mathcal{SB}_{\mathbb{E}}(\alpha)) \geq \mathbb{E}_{\mathfrak{S},s_0}(\mathcal{S}(\alpha))$.
- *A pair* $(\mathcal{RB}_{\mathbb{E}}, \mathcal{SB}_{\mathbb{E}})$ *is called an* expected bound pair.

*Example 9 (Expected Runtime and Size Bounds). Our new techniques from Sect. 4 and 5 will derive the following expected bounds for the PIP from Fig. 1.*

$$\mathcal{RB}_{\mathbb{E}}(g) = \begin{cases} 1, & \text{if } g \in \{g_0, g_2\} \\ 2\cdot x, & \text{if } g = g_1 \\ 6\cdot x^2 + 2\cdot y, & \text{if } g = g_3 \end{cases} \qquad \mathcal{SB}_{\mathbb{E}}(g, \_, x) = \begin{cases} x, & \text{if } g = g_0 \\ 2\cdot x, & \text{if } g = g_1 \\ 3\cdot x, & \text{if } g \in \{g_2, g_3\} \end{cases}$$

$$\mathcal{SB}_{\mathbb{E}}(g_0,\ell_1,y) = y \qquad \mathcal{SB}_{\mathbb{E}}(g_2,\ell_2,y) = 6\cdot x^2 + 2\cdot y$$

$$\mathcal{SB}_{\mathbb{E}}(g_1,\ell_1,y) = 6\cdot x^2 + y \qquad \mathcal{SB}_{\mathbb{E}}(g_3,\ell_2,y) = 12\cdot x^2 + 4\cdot y$$

*While the runtimes of* $t_2$ *and* $t_4$ *were unbounded in the non-probabilistic case (Ex. 5), we obtain finite bounds on the expected runtimes of* $g_1 = \{t_1, t_2\}$ *and* $g_3 = \{t_4\}$*. For example, we can expect* $x$ *to be non-positive after at most* $|s_0|\,(2\cdot x)$ *iterations of* $g_1$*. Based on the above expected runtime bounds, the expected runtime complexity of the PIP is at most* $|s_0|\,(\mathcal{RB}_{\mathbb{E}}(g_0) + \ldots + \mathcal{RB}_{\mathbb{E}}(g_3)) = |s_0|\,(2 + 2\cdot x + 2\cdot y + 6\cdot x^2)$*, i.e., it is in* $\mathcal{O}(n^2)$ *where* $n$ *is the maximal absolute value of the program variables at the start of the program.*

The following theorem shows that non-probabilistic bounds can be lifted to expected bounds, since they do not only bound the expected value of $\mathcal{R}(g)$ resp. $\mathcal{S}(\alpha)$, but the whole distribution. As mentioned, all proofs can be found in [47].

**Theorem 10 (Lifting Bounds).** *For a bound pair* $(\mathcal{RB}, \mathcal{SB})$*,* $(\mathcal{RB}_{\mathbb{E}}, \mathcal{SB}_{\mathbb{E}})$ *with* $\mathcal{RB}_{\mathbb{E}}(g) = \sum_{t\in g} \mathcal{RB}(t)$ *and* $\mathcal{SB}_{\mathbb{E}}(g,\ell,x) = \sum_{t=(\_,\_,\_,\_,\ell)\in g} \mathcal{SB}(t,x)$ *is an expected bound pair.*

Here, we over-approximate the maximum of $\mathcal{SB}(t,x)$ for $t = (\_,\_,\_,\_,\ell) \in g$ by their sum. For asymptotic bounds, this does not affect precision, since $\max(f,g)$ and $f+g$ have the same asymptotic growth for any non-negative functions $f, g$.

*Example 11 (Lifting of Bounds). When lifting the bound pair of Ex. 5 to expected bounds according to Thm. 10, one would obtain* $\mathcal{RB}_{\mathbb{E}}(g_0) = \mathcal{RB}_{\mathbb{E}}(g_2) = 1$ *and* $\mathcal{RB}_{\mathbb{E}}(g_1) = \mathcal{RB}_{\mathbb{E}}(g_3) = \infty$*. Moreover,* $\mathcal{SB}_{\mathbb{E}}(g_0,\ell_1,x) = x$*,* $\mathcal{SB}_{\mathbb{E}}(g_1,\ell_1,x) = 2\cdot x$*,* $\mathcal{SB}_{\mathbb{E}}(g_2,\ell_2,x) = \mathcal{SB}_{\mathbb{E}}(g_3,\ell_2,x) = 3\cdot x$*,* $\mathcal{SB}_{\mathbb{E}}(g_0,\ell_1,y) = y$*, and* $\mathcal{SB}_{\mathbb{E}}(g,\_,y) = \infty$ *whenever* $g \neq g_0$*. Thus, with these lifted bounds one cannot show that* $\mathcal{P}$*'s expected runtime complexity is finite, i.e., they are substantially less precise than the finite expected bounds from Ex. 9. Our approach will compute such finite expected bounds by repeatedly improving the lifted bounds of Thm. 10.*

## 4 Computing Expected Runtime Bounds

We first present a new variant of probabilistic linear ranking functions in Sect. 4.1. Based on this, in Sect. 4.2 we introduce our modular technique to infer expected runtime bounds by using expected size bounds.

### 4.1 Probabilistic Linear Ranking Functions

For probabilistic programs, several techniques based on ranking supermartingales have been developed. In this section, we define a class of probabilistic ranking functions that will be suitable for our modular analysis.

We restrict ourselves to ranking functions $\mathfrak{r} : \mathcal{L} \to \mathbb{R}[\mathcal{PV}]_{\text{lin}}$ that map every location to a *linear polynomial* (i.e., of at most degree 1) without temporary variables. The linearity restriction is common to ease the automated inference of ranking functions. Moreover, this restriction will be needed for the soundness of our technique. Nevertheless, our approach of course also infers non-linear expected runtimes (by combining the linear bounds obtained for different program parts).

Let $\exp_{\mathfrak{r},g,s}$ denote the expected value of $\mathfrak{r}$ after an execution of $g \in \mathcal{GT}$ in state $s \in \Sigma$. Here, $s_\eta(x)$ is the expected value of $x \in \mathcal{PV}$ after performing the update $\eta$ in state $s$. So if $\eta(x) \in \mathcal{D}$, then $x$'s expected value after the update results from adding the expected value of the probability distribution $\eta(x)(s)$:

$$\exp_{\mathfrak{r},g,s} = \sum_{(\ell,p,\tau,\eta,\ell')\in g} p \cdot s_\eta(\mathfrak{r}(\ell')) \text{ with } s_\eta(x) = \begin{cases} s(\eta(x)), & \text{if } \eta(x) \in \mathbb{Z}[\mathcal{V}] \\ s(x) + \mathbb{E}(\eta(x)(s)), & \text{if } \eta(x) \in \mathcal{D} \end{cases}$$

**Definition 12 (PLRF).** *Let $\mathcal{GT}_> \subseteq \mathcal{GT}_{\text{ni}} \subseteq \mathcal{GT}$. Then $\mathfrak{r} \colon \mathcal{L} \to \mathbb{R}[\mathcal{PV}]_{\text{lin}}$ is a* probabilistic linear ranking function (PLRF) *for $\mathcal{GT}_>$ and $\mathcal{GT}_{\text{ni}}$ if for all $g \in \mathcal{GT}_{\text{ni}} \setminus \mathcal{GT}_>$ and $c' \in \mathsf{Conf}$ there is a $\bowtie_{g,c'} \in \{<, \geq\}$ such that for all finite paths $\cdots c'\, c$ that are admissible for some $\mathfrak{S}$ and $s_0 \in \Sigma$, and where $c = (\ell, t, s)$ (i.e., where $t$ is the transition that is used in the step from $c'$ to $c$), we have:*
***Boundedness (a):*** *If $t \in g$ for a $g \in \mathcal{GT}_{\text{ni}} \setminus \mathcal{GT}_>$, then $s(\mathfrak{r}(\ell)) \bowtie_{g,c'} 0$.*
***Boundedness (b):*** *If $t \in g$ for a $g \in \mathcal{GT}_>$, then $s(\mathfrak{r}(\ell)) \geq 0$.*
***Non-Increase:*** *If $\ell = \ell_g$ for a $g \in \mathcal{GT}_{\text{ni}}$ and $s(\tau_g) = \mathbf{t}$, then $s(\mathfrak{r}(\ell)) \geq \exp_{\mathfrak{r},g,s}$.*
***Decrease:*** *If $\ell = \ell_g$ for a $g \in \mathcal{GT}_>$ and $s(\tau_g) = \mathbf{t}$, then $s(\mathfrak{r}(\ell)) - 1 \geq \exp_{\mathfrak{r},g,s}$.*

So if one is restricted to the sub-program with the non-increasing transitions $\mathcal{GT}_{\text{ni}}$, then $\mathfrak{r}(\ell)$ is an upper bound on the expected number of applications of transitions from $\mathcal{GT}_>$ when starting in $\ell$. Hence, a PLRF for $\mathcal{GT}_> = \mathcal{GT}_{\text{ni}} = \mathcal{GT}$ would imply that the program is PAST (see, e.g., [1,16,24,25]). However, our PLRFs differ from the standard notion of probabilistic ranking functions by considering arbitrary subsets $\mathcal{GT}_{\text{ni}} \subseteq \mathcal{GT}$. This is needed for the modularity of our approach which allows us to analyze program parts separately (e.g., $\mathcal{GT} \setminus \mathcal{GT}_{\text{ni}}$ is ignored when inferring a PLRF). Thus, our "Boundedness" conditions differ slightly from the corresponding conditions in other definitions. Condition (b) requires that $g \in \mathcal{GT}_>$ never leads to a configuration where $\mathfrak{r}$ is negative. Condition (a) states that in an admissible path where $g = \{t_1, t_2, \ldots\} \in \mathcal{GT}_{\text{ni}} \setminus \mathcal{GT}_>$ is used for continuing in configuration $c'$, if executing $t_1$ in $c'$ makes $\mathfrak{r}$ negative, then executing $t_2$ must make $\mathfrak{r}$ negative as well. Thus, such a $g$ can never come before a general transition from $\mathcal{GT}_>$ in an admissible path and hence, $g$ can be ignored when inferring upper bounds on the runtime. This increases the power of our approach and it allows us to consider only *non-negative* random variables in our correctness proofs.

We use SMT solvers to generate PLRFs automatically. Then for "Boundedness", we regard all $s' \in \Sigma$ with $s'(\tau_g) = \mathbf{t}$ and require "Boundedness" for any state $s$ that is reachable from $s'$.

*Example 13 (PLRFs). Consider again the PIP in Fig. 1 and the sets* $\mathcal{GT}_> = \mathcal{GT}_{\text{ni}} = \{g_1\}$ *and* $\mathcal{GT}'_> = \mathcal{GT}'_{\text{ni}} = \{g_3\}$*, which correspond to its two loops.*

*The function* $\mathfrak{r}$ *with* $\mathfrak{r}(\ell_1) = 2 \cdot x$ *and* $\mathfrak{r}(\ell_0) = \mathfrak{r}(\ell_2) = 0$ *is a PLRF for* $\mathcal{GT}_> = \mathcal{GT}_{\text{ni}}$*: For every admissible configuration* $(\ell, t, s)$ *with* $t \in g_1$ *we have* $\ell = \ell_1$ *and* $s(\mathfrak{r}(\ell_1)) = 2 \cdot s(x) \geq 0$*, since* $x$ *was positive before (due to* $g_1$*'s guard) and it was either decreased by* 1 *or not changed by the update of* $t_1$ *resp.* $t_2$*. Hence* $\mathfrak{r}$ *is bounded. Moreover, for* $s_1(x) = s(x-1) = s(x) - 1$ *and* $s_2(x) = s(x)$ *we have:*

$$\exp_{\mathfrak{r},g,s} \;=\; \tfrac{1}{2} \cdot s_1(\mathfrak{r}(\ell_1)) + \tfrac{1}{2} \cdot s_2(\mathfrak{r}(\ell_1)) \;=\; 2 \cdot s(x) - 1 \;=\; s(\mathfrak{r}(\ell_1)) - 1$$

*So* $\mathfrak{r}$ *is decreasing on* $g_1$ *and as* $\mathcal{GT}_> = \mathcal{GT}_{\text{ni}}$*, also the non-increase property holds. Similarly,* $\mathfrak{r}'$ *with* $\mathfrak{r}'(\ell_2) = y$ *and* $\mathfrak{r}'(\ell_0) = \mathfrak{r}'(\ell_1) = 0$ *is a PLRF for* $\mathcal{GT}'_> = \mathcal{GT}'_{\text{ni}}$*.*

In our implementation, $\mathcal{GT}_>$ is always a singleton and we let $\mathcal{GT}_{\text{ni}} \subseteq \mathcal{GT}$ be a cycle in the call graph where we find a PLRF for $\mathcal{GT}_> \subseteq \mathcal{GT}_{\text{ni}}$. The next subsection shows how we can then obtain an expected runtime bound for the overall program by searching for suitable ranking functions repeatedly.

### 4.2 Inferring Expected Runtime Bounds

Our approach to infer expected runtime bounds is based on an underlying (non-probabilistic) bound pair $(\mathcal{RB}, \mathcal{SB})$ which is computed by existing techniques (in our implementation, we use [18]). To do so, we abstract the PIP to a standard integer transition system by ignoring the probabilities of transitions and replacing probabilistic with non-deterministic sampling (e.g., the update $\eta(x) = \text{GEO}(\frac{1}{2})$ would be replaced by $\eta(x) = x + u$ with $u \in \mathcal{V} \setminus \mathcal{PV}$, where $u > 0$ is added to the guard). Of course, we usually have $\mathcal{RB}(t) = \infty$ for some transitions $t$.

We start with the expected bound pair $(\mathcal{RB}_{\mathbb{E}}, \mathcal{SB}_{\mathbb{E}})$ that is obtained by lifting $(\mathcal{RB}, \mathcal{SB})$ as in Thm. 10. Afterwards, the expected runtime bound $\mathcal{RB}_{\mathbb{E}}$ is improved repeatedly by applying the following Thm. 16 (and similarly, $\mathcal{SB}_{\mathbb{E}}$ is improved repeatedly by applying Thm. 23 and 25 from Sect. 5). Our approach alternates the improvement of $\mathcal{RB}_{\mathbb{E}}$ and $\mathcal{SB}_{\mathbb{E}}$, and it uses expected size bounds on "previous" transitions to improve expected runtime bounds, and vice versa.

To improve $\mathcal{RB}_{\mathbb{E}}$, we generate a PLRF $\mathfrak{r}$ for a part of the program. To obtain a bound for the *full* program from $\mathfrak{r}$, one has to determine which transitions can enter the program part and from which locations it can be entered.

**Definition 14 (Entry Locations and Transitions).** *For* $\mathcal{GT}_{\text{ni}} \subseteq \mathcal{GT}$ *and* $\ell \in \mathcal{L}$*, the* entry transitions *are* $\mathcal{ET}_{\mathcal{GT}_{\text{ni}}}(\ell) = \{g \in \mathcal{GT} \setminus \mathcal{GT}_{\text{ni}} \mid \exists t \in g.\, t = (\_,\_,\_,\_,\ell)\}$*. Then the* entry locations *are all start locations of* $\mathcal{GT}_{\text{ni}}$ *whose entry transitions*

*are not empty, i.e.,* $\mathcal{EL}_{\mathcal{GT}_{\mathrm{ni}}} = \{\ell \mid \mathcal{ET}_{\mathcal{GT}_{\mathrm{ni}}}(\ell) \neq \varnothing \wedge (\ell, \_, \_, \_, \_) \in \bigcup \mathcal{GT}_{\mathrm{ni}}\}$.[1]

*Example 15 (Entry Locations and Transitions). For the PIP from Fig. 1 and* $\mathcal{GT}_{\mathrm{ni}} = \{g_1\}$*, we have* $\mathcal{EL}_{\mathcal{GT}_{\mathrm{ni}}} = \{\ell_1\}$ *and* $\mathcal{ET}_{\mathcal{GT}_{\mathrm{ni}}}(\ell_1) = \{g_0\}$*. So the loop formed by* $g_1$ *is entered at location* $\ell_1$ *and the general transition* $g_0$ *has to be executed before. Similarly, for* $\mathcal{GT}'_{\mathrm{ni}} = \{g_3\}$ *we have* $\mathcal{EL}_{\mathcal{GT}'_{\mathrm{ni}}} = \{\ell_2\}$ *and* $\mathcal{ET}_{\mathcal{GT}'_{\mathrm{ni}}}(\ell_2) = \{g_2\}$*.*

Recall that if $\mathfrak{r}$ is a PLRF for $\mathcal{GT}_> \subseteq \mathcal{GT}_{\mathrm{ni}}$, then in a program that is restricted to $\mathcal{GT}_{\mathrm{ni}}$, $\mathfrak{r}(\ell)$ is an upper bound on the expected number of executions of transitions from $\mathcal{GT}_>$ when starting in $\ell$. Since $\mathfrak{r}(\ell)$ may contain negative coefficients, it is not weakly monotonically increasing in general. To turn expressions $e \in \mathbb{R}[\mathcal{PV}]$ into bounds from $\mathcal{B}$, let the over-approximation $\lceil \cdot \rceil$ replace all coefficients by their absolute value. So for example, $\lceil x - y \rceil = \lceil x + (-1) \cdot y \rceil = x + y$. Clearly, we have $|s|\,(\lceil e \rceil) \geq |s|\,(e)$ for all $s \in \Sigma$. Moreover, if $e \in \mathbb{R}[\mathcal{PV}]$ then $\lceil e \rceil \in \mathcal{B}$.

To turn $\lceil \mathfrak{r}(\ell) \rceil$ into a bound for the full program, one has to take into account how often the sub-program with the transitions $\mathcal{GT}_{\mathrm{ni}}$ is reached via an entry transition $h \in \mathcal{ET}_{\mathcal{GT}_{\mathrm{ni}}}(\ell)$ for some $\ell \in \mathcal{EL}_{\mathcal{GT}_{\mathrm{ni}}}$. This can be over-approximated by $\sum_{t=(\_,\_,\_,\_,\ell)\in h} \mathcal{RB}(t)$, which is an upper bound on the number of times that transitions in $h$ to the entry location $\ell$ of $\mathcal{GT}_{\mathrm{ni}}$ are applied in a full program run.

The bound $\lceil \mathfrak{r}(\ell) \rceil$ is expressed in terms of the program variables at the entry location $\ell$ of $\mathcal{GT}_{\mathrm{ni}}$. To obtain a bound in terms of the variables at the start of the program, one has to take into account which value a program variable $x$ may have when the sub-program $\mathcal{GT}_{\mathrm{ni}}$ is reached. For every entry transition $h \in \mathcal{ET}_{\mathcal{GT}_{\mathrm{ni}}}(\ell)$, this value can be over-approximated by $\mathcal{SB}_{\mathbb{E}}(h, \ell, x)$. Thus, we have to instantiate each variable $x$ in $\lceil \mathfrak{r}(\ell) \rceil$ by $\mathcal{SB}_{\mathbb{E}}(h, \ell, x)$. Let $\mathcal{SB}_{\mathbb{E}}(h, \ell, \cdot) : \mathcal{PV} \to \mathcal{B}$ be the mapping with $\mathcal{SB}_{\mathbb{E}}(h, \ell, \cdot)(x) = \mathcal{SB}_{\mathbb{E}}(h, \ell, x)$. Hence, $\mathcal{SB}_{\mathbb{E}}(h, \ell, \cdot)(\lceil \mathfrak{r}(\ell) \rceil)$ over-approximates the expected number of applications of $\mathcal{GT}_>$ if $\mathcal{GT}_{\mathrm{ni}}$ is entered in location $\ell$, where this bound is expressed in terms of the input variables of the program. Here, weak monotonic increase of $\lceil \mathfrak{r}(\ell) \rceil$ ensures that instantiating its variables by an over-approximation of their size yields an over-approximation of the runtime.

**Theorem 16 (Expected Runtime Bounds).** *Let* $(\mathcal{RB}_{\mathbb{E}}, \mathcal{SB}_{\mathbb{E}})$ *be an expected bound pair,* $\mathcal{RB}$ *a (non-probabilistic) runtime bound, and* $\mathfrak{r}$ *a PLRF for* $\mathcal{GT}_> \subseteq \mathcal{GT}_{\mathrm{ni}} \subseteq \mathcal{GT}$*. Then* $\mathcal{RB}'_{\mathbb{E}} : \mathcal{GT} \to \mathcal{B}$ *is an expected runtime bound where*

$$\mathcal{RB}'_{\mathbb{E}}(g) = \begin{cases} \sum\limits_{\substack{\ell \in \mathcal{EL}_{\mathcal{GT}_{\mathrm{ni}}} \\ h \in \mathcal{ET}_{\mathcal{GT}_{\mathrm{ni}}}(\ell)}} \Big( \sum\limits_{t=(\_,\_,\_,\_,\ell)\in h} \mathcal{RB}(t) \Big) \cdot \big(\mathcal{SB}_{\mathbb{E}}(h, \ell, \cdot)\,(\lceil \mathfrak{r}(\ell) \rceil)\big), & \text{if } g \in \mathcal{GT}_> \\ \mathcal{RB}_{\mathbb{E}}(g), & \text{if } g \notin \mathcal{GT}_> \end{cases}$$

*Example 17 (Expected Runtime Bounds). For the PIP from Fig. 1, our approach starts with* $(\mathcal{RB}_{\mathbb{E}}, \mathcal{SB}_{\mathbb{E}})$ *from Ex. 11 which results from lifting the bound pair from Ex. 5. To improve the bound* $\mathcal{RB}_{\mathbb{E}}(g_1) = \infty$*, we use the PLRF* $\mathfrak{r}$ *for* $\mathcal{GT}_> = \mathcal{GT}_{\mathrm{ni}} = \{g_1\}$ *from Ex. 13. By Ex. 15, we have* $\mathcal{EL}_{\mathcal{GT}_{\mathrm{ni}}} = \{\ell_1\}$ *and* $\mathcal{ET}_{\mathcal{GT}_{\mathrm{ni}}}(\ell_1) = \{g_0\}$ *with* $g_0 = \{t_0\}$*, whose runtime bound is* $\mathcal{RB}(t_0) = 1$*, see Ex. 5. Using the expected size bound* $\mathcal{SB}_{\mathbb{E}}(g_0, \ell_1, x) = x$ *from Ex. 9, Thm. 16 yields*

$$\mathcal{RB}'_{\mathbb{E}}(g_1) = \mathcal{RB}(t_0) \cdot \mathcal{SB}_{\mathbb{E}}(g_0, \ell_1, \cdot)\,(\lceil \mathfrak{r}(\ell_1) \rceil) = 1 \cdot 2 \cdot x = 2 \cdot x.$$

[1] For a set of sets like $\mathcal{GT}_{\mathrm{ni}}$, $\bigcup \mathcal{GT}_{\mathrm{ni}}$ denotes their union, i.e., $\bigcup \mathcal{GT}_{\mathrm{ni}} = \bigcup_{g \in \mathcal{GT}_{\mathrm{ni}}} g$.

*To improve* $\mathcal{RB}_{\mathbb{E}}(g_3)$*, we use the PLRF* $\mathfrak{r}'$ *for* $\mathcal{GT}'_{>} = \mathcal{GT}'_{\mathrm{ni}} = \{g_3\}$ *from Ex. 13. As* $\mathcal{EL}_{\mathcal{GT}'_{\mathrm{ni}}} = \{\ell_2\}$ *and* $\mathcal{ET}_{\mathcal{GT}'_{\mathrm{ni}}}(\ell_2) = \{g_2\}$ *by Ex. 15, where* $g_2 = \{t_3\}$ *and* $\mathcal{RB}(t_3) = 1$ *(Ex. 5), with the bound* $\mathcal{SB}_{\mathbb{E}}(g_2, \ell_2, y) = 6 \cdot x^2 + 2 \cdot y$ *from Ex. 9, Thm. 16 yields*

$$\mathcal{RB}'_{\mathbb{E}}(g_3) \;=\; \mathcal{RB}(t_3) \cdot \mathcal{SB}_{\mathbb{E}}(g_2, \ell_2, \cdot)\,(\lceil \mathfrak{r}'(\ell_2) \rceil) = 1 \cdot \mathcal{SB}_{\mathbb{E}}(g_2, \ell_2, y) = 6 \cdot x^2 + 2 \cdot y.$$

*So based on the expected size bounds of Ex. 9, we have shown how to compute the expected runtime bounds of Ex. 9 automatically.*

Similar to [18], our approach relies on combining bounds that one has computed earlier in order to derive new bounds. Here, bounds may be combined *linearly*, bounds may be *multiplied*, and bounds may even be *substituted* into other bounds. But in contrast to [18], sometimes one may combine *expected* bounds that were computed earlier and sometimes it is only sound to combine *non-probabilistic* bounds: If a new bound is computed by *linear combinations* of earlier bounds, then it is sound to use the "expected versions" of these earlier bounds. However, if two bounds are *multiplied*, then it is in general not sound to use their "expected versions". Thus, it would be *unsound* to use the *expected* runtime bounds $\mathcal{RB}_{\mathbb{E}}(h)$ instead of the *non-probabilistic* bounds $\sum_{t=(\_,\_,\_,\_,\ell)\in h} \mathcal{RB}(t)$ on the entry transitions in Thm. 16 (a counterexample is given in [47]).[2]

In general, if bounds $b_1, \ldots, b_n$ are *substituted* into another bound $b$, then it is sound to use "expected versions" of the bounds $b_1, \ldots, b_n$ if $b$ is *concave*, see, e.g., [10, 11, 40]. Since bounds from $\mathcal{B}$ do not contain negative coefficients, we obtain that a finite[3] bound $b \in \mathcal{B}$ is concave iff it is a linear polynomial (see [47]).

Thus, in Thm. 16 we may substitute *expected* size bounds $\mathcal{SB}_{\mathbb{E}}(h, \ell, x)$ into $\lceil \mathfrak{r}(\ell) \rceil$, since we restricted ourselves to *linear* ranking functions $\mathfrak{r}$ and hence, $\lceil \mathfrak{r}(\ell) \rceil$ is also linear. Note that in contrast to [11], where a notion of concavity was used to analyze probabilistic term rewriting, a multilinear expression like $x \cdot y$ is not concave when regarding both arguments simultaneously. Hence, it is unsound to use such ranking functions in Thm. 16. See [47] for a counterexample to show why substituting expected bounds into a non-linear bound is incorrect in general.

## 5 Computing Expected Size Bounds

We first compute *local* bounds for one application of a transition (Sect. 5.1). To turn them into *global* bounds, we encode the data flow of a PIP in a graph. Sect. 5.2 then presents our technique to compute expected size bounds.

### 5.1 Local Change Bounds and General Result Variable Graph

We first compute a bound on the expected change of a variable during an update. More precisely, for every general result variable $(g, \ell, x)$ we define a bound $\mathcal{CB}_{\mathbb{E}}(g, \ell, x)$ on the change of the variable $x$ that we can expect in one

2 An exception is the special case where $\mathfrak{r}(\ell)$ is *constant*. Then, our implementation indeed uses the expected bound $\mathcal{RB}_{\mathbb{E}}(h)$ instead of $\sum_{t=(\_,\_,\_,\_,\ell)\in h} \mathcal{RB}(t)$ [47].

3 A bound is *finite* if it does not contain $\infty$. We always simplify expressions and thus, a bound like $0 \cdot \infty$ is also finite, because it simplifies to 0, as usual in measure theory.

execution of the general transition $g$ when reaching location $\ell$. So we consider all $t = (\_, p, \_, \eta, \ell) \in g$ and the expected difference between the current value of $x$ and its update $\eta(x)$. However, for $\eta(x) \in \mathbb{Z}[\mathcal{V}]$, $\eta(x) - x$ is not necessarily from $\mathcal{B}$ because it may contain negative coefficients. Thus, we use the over-approximation $\lceil \eta(x) - x \rceil$ (where we always simplify expressions before applying $\lceil \cdot \rceil$, e.g., $\lceil x - x \rceil = \lceil 0 \rceil = 0$). Moreover, $\lceil \eta(x) - x \rceil$ may contain temporary variables. Let $\mathrm{tv}_t : \mathcal{V} \to \mathcal{B}$ instantiate all temporary variables by the largest possible value they can have after evaluating the transition $t$. Hence, we then use $\mathrm{tv}_t(\lceil \eta(x) - x \rceil)$ instead. For $\mathrm{tv}_t$, we have to use the underlying *non-probabilistic* size bound $\mathcal{SB}$ for the program (since the scheduler determines the values of temporary variables by non-deterministic (*non-probabilistic*) choice). If $x$ is updated according to a bounded distribution function $d \in \mathcal{D}$, then as in Sect. 2, let $\mathfrak{E}(d) \in \mathcal{B}$ denote a finite bound on $d$, i.e., $\mathbb{E}_{\mathrm{abs}}(d(s)) \leq |s|\,(\mathfrak{E}(d))$ for all $s \in \Sigma$.

**Definition 18 (Expected Local Change Bound).** *Let $\mathcal{SB}$ be a size bound. Then $\mathcal{CB}_{\mathbb{E}} \colon \mathcal{GRV} \to \mathcal{B}$ with $\mathcal{CB}_{\mathbb{E}}(g, \ell, x) = \sum_{t=(\_,p,\_,\eta,\ell)\in g} p \cdot \mathrm{ch}_t(\eta(x), x)$, where*

$$\mathrm{ch}_t(\eta(x), x) = \begin{cases} \mathfrak{E}(d), & \text{if } \eta(x) = d \in \mathcal{D} \\ \mathrm{tv}_t(\lceil \eta(x) - x \rceil), & \text{otherwise} \end{cases} \quad \text{and } \mathrm{tv}_t(y) = \begin{cases} \mathcal{SB}(t, y), & \text{if } y \notin \mathcal{PV} \\ y, & \text{if } y \in \mathcal{PV} \end{cases}$$

*Example 19 ($\mathcal{CB}_{\mathbb{E}}$). For the PIP of Fig. 1, we have $\mathcal{CB}_{\mathbb{E}}(g_0, \_, \_) = \mathcal{CB}_{\mathbb{E}}(g_2, \_, \_) = \mathcal{CB}_{\mathbb{E}}(g_3, \ell_2, x) = 0$, since the respective updates are identities. Moreover,*

$$\mathcal{CB}_{\mathbb{E}}(g_1, \ell_1, x) \;=\; \tfrac{1}{2} \cdot \lceil (x-1) - x \rceil + \tfrac{1}{2} \cdot \lceil x - x \rceil \;=\; \tfrac{1}{2} \cdot 1 + \tfrac{1}{2} \cdot 0 \;=\; \tfrac{1}{2}.$$

*In a similar way, we obtain $\mathcal{CB}_{\mathbb{E}}(g_1, \ell_1, y) = x$ and $\mathcal{CB}_{\mathbb{E}}(g_3, \ell_2, y) = 1$.*

The following theorem shows that for any admissible configuration in a state $s'$, $\mathcal{CB}_{\mathbb{E}}(g, \ell, x)$ is an upper bound on the expected value of $|s(x) - s'(x)|$ if $s$ is the next state obtained when applying $g$ in state $s'$ to reach location $\ell$.

**Theorem 20 (Soundness of $\mathcal{CB}_{\mathbb{E}}$).** *For any $(g, \ell, x) \in \mathcal{GRV}$, scheduler $\mathfrak{S}$, $s_0 \in \Sigma$, and admissible configuration $c' = (\_, \_, s')$, we have*

$$|s'|\,(\mathcal{CB}_{\mathbb{E}}(g, \ell, x)) \;\geq\; \sum\nolimits_{c=(\ell,t,s)\in \mathsf{Conf},\; t \in g} pr_{\mathfrak{S}, s_0}(c' \to c) \cdot |s(x) - s'(x)|.$$

To obtain *global* bounds from the local bounds $\mathcal{CB}_{\mathbb{E}}(g, \ell, x)$, we construct a *general result variable graph* which encodes the data flow between variables. Let $\mathrm{pre}(g) = \mathcal{ET}_{\varnothing}(\ell_g)$ be the the set of *pre-transitions* of $g$ which lead into $g$'s start location $\ell_g$. Moreover, for $\alpha = (g, \ell, x) \in \mathcal{GRV}$ let its *active variables* $\mathrm{actV}(\alpha)$ consist of all variables occurring in the bound $x + \mathcal{CB}_{\mathbb{E}}(\alpha)$ for $\alpha$'s expected size.

**Definition 21 (General Result Variable Graph).** *The* general result variable graph *has the set of nodes $\mathcal{GRV}$ and the set of edges $\mathcal{GRVE}$, where*

$$\mathcal{GRVE} = \{\, ((g', \ell', x'),\; (g, \ell, x)) \mid g' \in \mathrm{pre}(g) \wedge \ell' = \ell_g \wedge x' \in \mathrm{actV}(g, \ell, x) \,\}.$$

*Example 22 (General Result Variable Graph). The general result variable graph for the PIP of Fig. 1 is shown below. For $\mathcal{CB}_{\mathbb{E}}$ from Ex. 19, we have $\mathrm{actV}(g_1, \ell_1, x) = \{x\}$, as $x + \mathcal{CB}_{\mathbb{E}}(\alpha) = x + \frac{1}{2}$ contains no variable except $x$.*

*Similarly,* $\mathrm{actV}(g_1, \ell_1, y) = \{x, y\}$*, as* $x$ *and* $y$ *are contained in* $y + \mathcal{CB}_{\mathbb{E}}(g_1, \ell_1, y) = y + x$*. For all other* $\alpha \in \mathcal{GRV}$*, we have* $\mathrm{actV}(\_, \_, x) = \{x\}$ *and* $\mathrm{actV}(\_, \_, y) = \{y\}$*. As* $\mathrm{pre}(g_1) = \{g_0, g_1\}$*, the graph captures the dependence of* $(g_1, \ell_1, x)$ *on* $(g_0, \ell_1, x)$ *and* $(g_1, \ell_1, x)$*, and of* $(g_1, \ell_1, y)$ *on* $(g_0, \ell_1, x)$*,* $(g_0, \ell_1, y)$*,* $(g_1, \ell_1, x)$*, and* $(g_1, \ell_1, y)$*. The other edges are obtained in a similar way.*

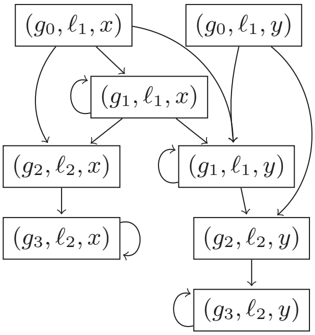

### 5.2 Inferring Expected Size Bounds

We now compute global expected size bounds for the general result variables by considering the SCCs of the general result variable graph separately. As usual, an SCC is a maximal subgraph with a path from each node to every other node. An SCC is *trivial* if it consists of a single node without an edge to itself. We first handle trivial SCCs in Sect. 5.2.1 and consider non-trivial SCCs in Sect. 5.2.2.

**5.2.1 Inferring Expected Size Bounds for Trivial SCCs** By Thm. 20, $x + \mathcal{CB}_{\mathbb{E}}(g, \ell, x)$ is a *local* bound on the expected value of $x$ after applying $g$ once in order to enter $\ell$. However, this bound is formulated in terms of the values of the variables immediately before applying $g$. We now want to compute *global* bounds in terms of the *initial* values of the variables at the start of the program.

If $g$ is *initial* (i.e., $g \in \mathcal{GT}_0$ since $g$ starts in the initial location $\ell_0$), then $x + \mathcal{CB}_{\mathbb{E}}(g, \ell, x)$ is already a global bound, as the values of the variables before the application of $g$ are the initial values of the variables at the program start.

Otherwise, the variables $y$ occurring in the local bound $x + \mathcal{CB}_{\mathbb{E}}(g, \ell, x)$ have to be replaced by the values that they can take in a full program run before applying the transition $g$. Thus, we have to consider all transitions $h \in \mathrm{pre}(g)$ and instantiate every variable $y$ by the maximum of the values that $y$ can have after applying $h$. Here, we again over-approximate the maximum by the sum.

If $\mathcal{CB}_{\mathbb{E}}(g, \ell, x)$ is *concave* (i.e., a *linear* polynomial), then we can instantiate its variables by *expected* size bounds $\mathcal{SB}_{\mathbb{E}}(h, \ell_g, y)$. However, this is unsound if $\mathcal{CB}_{\mathbb{E}}(g, \ell, x)$ is not linear, i.e., not concave (see [47] for a counterexample). So in this case, we have to use *non-probabilistic* bounds $\mathcal{SB}(t, y)$ instead.

As in Sect. 4.2, we use an underlying non-probabilistic bound pair $(\mathcal{RB}, \mathcal{SB})$ and start with the expected pair $(\mathcal{RB}_{\mathbb{E}}, \mathcal{SB}_{\mathbb{E}})$ obtained by lifting $(\mathcal{RB}, \mathcal{SB})$ according to Thm. 10. While Thm. 16 improves $\mathcal{RB}_{\mathbb{E}}$, we now improve $\mathcal{SB}_{\mathbb{E}}$. Here, the SCCs of the general result variable graph should be treated in topological order, since then one may first improve $\mathcal{SB}_{\mathbb{E}}$ for result variables corresponding to $\mathrm{pre}(g)$, and use that when improving $\mathcal{SB}_{\mathbb{E}}$ for result variables of the form $(g, \_, \_)$.

**Theorem 23 (Expected Size Bounds for Trivial SCCs).** *Let* $\mathcal{SB}_{\mathbb{E}}$ *be an expected size bound,* $\mathcal{SB}$ *a (non-probabilistic) size bound, and let* $\alpha = (g, \ell, x)$ *form a trivial SCC of the general result variable graph. Let* $\mathrm{size}^{\alpha}_{\mathbb{E}}$ *and* $\mathrm{size}^{\alpha}$ *be mappings from* $\mathcal{PV} \to \mathcal{B}$ *with* $\mathrm{size}^{\alpha}_{\mathbb{E}}(y) = \sum_{h \in \mathrm{pre}(g)} \mathcal{SB}_{\mathbb{E}}(h, \ell_g, y)$ *and* $\mathrm{size}^{\alpha}(y) = \sum_{h \in \mathrm{pre}(g),\, t = (\_,\_,\_,\_,\ell_g) \in h} \mathcal{SB}(t, y)$*. Then* $\mathcal{SB}'_{\mathbb{E}} : \mathcal{GRV} \to \mathcal{B}$ *is an expected size bound,*

*where* $\mathcal{SB}'_{\mathbb{E}}(\beta) = \mathcal{SB}_{\mathbb{E}}(\beta)$ *for* $\beta \neq \alpha$ *and*

$$\mathcal{SB}'_{\mathbb{E}}(\alpha) = \begin{cases} x + \mathcal{CB}_{\mathbb{E}}(\alpha), & \text{if } g \in \mathcal{GT}_0 \\ \text{size}^{\alpha}_{\mathbb{E}}(x + \mathcal{CB}_{\mathbb{E}}(\alpha)), & \text{if } g \notin \mathcal{GT}_0,\ \mathcal{CB}_{\mathbb{E}}(\alpha) \text{ is linear} \\ \text{size}^{\alpha}_{\mathbb{E}}(x) + \text{size}^{\alpha}(\mathcal{CB}_{\mathbb{E}}(\alpha)), & \text{if } g \notin \mathcal{GT}_0,\ \mathcal{CB}_{\mathbb{E}}(\alpha) \text{ is not linear} \end{cases}$$

*Example 24 ($\mathcal{SB}_{\mathbb{E}}$ for Trivial SCCs). The general result variable graph in Ex. 22 contains 4 trivial SCCs formed by $\alpha_x = (g_0, \ell_1, x)$, $\alpha_y = (g_0, \ell_1, y)$, $\beta_x = (g_2, \ell_2, x)$, and $\beta_y = (g_2, \ell_2, y)$. For all these general result variables, the expected local change bound $\mathcal{CB}_{\mathbb{E}}$ is 0 (see Ex. 19). Thus, it is linear. Since $g_0 \in \mathcal{GT}_0$, Thm. 23 yields $\mathcal{SB}'_{\mathbb{E}}(\alpha_x) = x + \mathcal{CB}_{\mathbb{E}}(\alpha_x) = x$ and $\mathcal{SB}'_{\mathbb{E}}(\alpha_y) = y + \mathcal{CB}_{\mathbb{E}}(\alpha_y) = y$.*

*By treating SCCs in topological order, when handling $\beta_x$, $\beta_y$, we can assume that we already have $\mathcal{SB}_{\mathbb{E}}(\alpha_x) = x$, $\mathcal{SB}_{\mathbb{E}}(\alpha_y) = y$ and $\mathcal{SB}_{\mathbb{E}}(g_1, \ell_1, x) = 2 \cdot x$, $\mathcal{SB}_{\mathbb{E}}(g_1, \ell_1, y) = 6 \cdot x^2 + y$ (see Ex. 9) for the result variables corresponding to* $\text{pre}(g_2) = \{g_0, g_1\}$. *We will explain in Sect. 5.2.2 how to compute such expected size bounds for non-trivial SCCs. Hence, by Thm. 23 we obtain $\mathcal{SB}'_{\mathbb{E}}(\beta_x) = \text{size}^{\beta_x}_{\mathbb{E}}(x + \mathcal{CB}_{\mathbb{E}}(\beta_x)) = \mathcal{SB}_{\mathbb{E}}(\alpha_x) + \mathcal{SB}_{\mathbb{E}}(g_1, \ell_1, x) = 3 \cdot x$ and $\mathcal{SB}'_{\mathbb{E}}(\beta_y) = \text{size}^{\beta_y}_{\mathbb{E}}(y + \mathcal{CB}_{\mathbb{E}}(\beta_y)) = \mathcal{SB}_{\mathbb{E}}(\alpha_y) + \mathcal{SB}_{\mathbb{E}}(g_1, \ell_1, y) = 6 \cdot x^2 + 2 \cdot y$.*

**5.2.2 Inferring Expected Size Bounds for Non-Trivial SCCs** Now we handle non-trivial SCCs $C$ of the general result variable graph. An upper bound for the expected size of a variable $x$ when entering $C$ is obtained from $\mathcal{SB}_{\mathbb{E}}(\beta)$ for all general result variables $\beta = (\_, \_, x)$ which have an edge to $C$.

To turn $\mathcal{CB}_{\mathbb{E}}(g, \ell, x)$ into a global bound, as in Thm. 23 its variables $y$ have to be instantiated by the values $\text{size}^{(g,\ell,x)}(y)$ that they can take in a full program run before applying a transition from $g$. Thus, $\text{size}^{(g,\ell,x)}(\mathcal{CB}_{\mathbb{E}}(g, \ell, x))$ is a global bound on the expected change resulting from *one* application of $g$. To obtain an upper bound for the whole SCC $C$, we add up these global bounds for all $(g, \_, x) \in C$ and take into account how often the general transitions in the SCC are expected to be executed, i.e., we multiply with their expected runtime bound $\mathcal{RB}_{\mathbb{E}}(g)$. So while in Thm. 16 we improve $\mathcal{RB}_{\mathbb{E}}$ using expected size bounds for previous transitions, we now improve $\mathcal{SB}_{\mathbb{E}}(C)$ using expected runtime bounds for the transitions in $C$ and expected size bounds for previous transitions.

**Theorem 25 (Expected Size Bounds for Non-Trivial SCCs).** *Let $(\mathcal{RB}_{\mathbb{E}}, \mathcal{SB}_{\mathbb{E}})$ be an expected bound pair, $(\mathcal{RB}, \mathcal{SB})$ a (non-probabilistic) bound pair, and let $C \subseteq \mathcal{GRV}$ form a non-trivial SCC of the general result variable graph where $\mathcal{GT}_C = \{g \in \mathcal{GT} \mid (g, \_, \_) \in C\}$. Then $\mathcal{SB}'_{\mathbb{E}}$ is an expected size bound:*

$$\mathcal{SB}'_{\mathbb{E}}(\alpha) = \begin{cases} \sum_{(\beta,\alpha)\in\mathcal{GRVE},\ \beta\notin C,\ \alpha\in C,\ \beta=(\_,\_,x)} \mathcal{SB}_{\mathbb{E}}(\beta) \quad + \\ \quad \sum\limits_{g\in\mathcal{GT}_C} \mathcal{RB}_{\mathbb{E}}(g) \cdot \Big( \sum\limits_{\alpha'=(g,\_,x)\in C} \text{size}^{\alpha'}(\mathcal{CB}_{\mathbb{E}}(\alpha')) \Big), & \text{if } \alpha = (\_, \_, x) \in C \\ \mathcal{SB}_{\mathbb{E}}(\alpha), & \text{otherwise} \end{cases}$$

Here we really have to use the *non-probabilistic* size bound $\text{size}^{\alpha'}$ instead of $\text{size}^{\alpha'}_{\mathbb{E}}$, even if $\mathcal{CB}_{\mathbb{E}}(\alpha')$ is linear, i.e., concave. Otherwise we would multiply the expected values of two random variables which are not independent.

*Example 26 ($\mathcal{SB}_{\mathbb{E}}$ for Non-Trivial SCCs). The general result variable graph in*

*Ex. 22 contains 4 non-trivial SCCs formed by* $\alpha'_x = (g_1, \ell_1, x)$, $\alpha'_y = (g_1, \ell_1, y)$, $\beta'_x = (g_3, \ell_2, x)$, *and* $\beta'_y = (g_3, \ell_2, y)$. *By the results on* $\mathcal{SB}_\mathbb{E}$, $\mathcal{RB}_\mathbb{E}$, $\mathcal{CB}_\mathbb{E}$, *and* $\mathcal{SB}$ *from Ex. 24, 17, 19, and 5, Thm. 25 yields the expected size bound in Ex. 9:*

$$\begin{aligned}
\mathcal{SB}'_\mathbb{E}(\alpha'_x) &= \mathcal{SB}_\mathbb{E}(\alpha_x) + \mathcal{RB}_\mathbb{E}(g_1) \cdot \mathrm{size}^{\alpha'_x}(\mathcal{CB}_\mathbb{E}(\alpha'_x)) &&= x + 2 \cdot x \cdot \tfrac{1}{2} = 2 \cdot x \\
\mathcal{SB}'_\mathbb{E}(\alpha'_y) &= \mathcal{SB}_\mathbb{E}(\alpha_y) + \mathcal{RB}_\mathbb{E}(g_1) \cdot \mathrm{size}^{\alpha'_y}(\mathcal{CB}_\mathbb{E}(\alpha'_y)) &&= y + 2 \cdot x \cdot \mathrm{size}^{\alpha'_y}(x) \\
&= y + 2 \cdot x \cdot \textstyle\sum_{i \in \{0,1,2\}} \mathcal{SB}(t_i, x) &&= 6 \cdot x^2 + y \\
\mathcal{SB}'_\mathbb{E}(\beta'_x) &= \mathcal{SB}_\mathbb{E}(\beta_x) + \mathcal{RB}_\mathbb{E}(g_3) \cdot \mathrm{size}^{\beta'_x}(\mathcal{CB}_\mathbb{E}(\beta'_x)) &&= 3 \cdot x + (6x^2 + 2y) \cdot 0 = 3 \cdot x \\
\mathcal{SB}'_\mathbb{E}(\beta'_y) &= \mathcal{SB}_\mathbb{E}(\beta_y) + \mathcal{RB}_\mathbb{E}(g_3) \cdot \mathrm{size}^{\beta'_y}(\mathcal{CB}_\mathbb{E}(\beta'_y)) &&= 6 \cdot x^2 + 2 \cdot y + (6x^2 + 2y) \cdot 1 \\
& &&= 12 \cdot x^2 + 4 \cdot y
\end{aligned}$$

## 6 Related Work, Implementation, and Conclusion

*Related Work* Our approach adapts techniques from [18] to probabilistic programs. As explained in Sect. 1, this adaption is not at all trivial (see our proofs in [47]).

There has been a lot of work on proving PAST and inferring bounds on expected runtimes using supermartingales, e.g., [1,11,15,16,22–25,29,32,48,62]. While these techniques infer one (lexicographic) ranking supermartingale to analyze the complete program, our approach deals with information flow between different program parts and analyzes them separately.

There is also work on modular analysis of almost sure termination (AST) [1,25,26,37,38,48], i.e., termination with probability 1. This differs from our results, since AST is compositional, in contrast to PAST (see, e.g., [41,42]).

A fundamentally different approach to ranking supermartingales (i.e., to *forward-reasoning*) is *backward-reasoning* by so-called expectation transformers, see, e.g., [10,41,42,44–46,50,52,61]. In this orthogonal reasoning, [10,41,42,52] consider the connection of the expected runtime and size. While expectation transformers apply backward- instead of forward-reasoning, their correctness can also be justified using supermartingales. More precisely, Park induction for upper bounds on the expected runtime via expectation transformers essentially ensures that a certain stochastic process is a supermartingale (see [33] for details).

To the best of our knowledge, the only available tools for the inference of upper bounds on the expected runtimes of probabilistic programs are [10,50,61,62]. The tool of [61] deals with data types and higher order functions in probabilistic ML programs and does not support programs whose complexity depends on (possibly negative) integers (see [55]). Furthermore, the tool of [48] focuses on proving or refuting (P)AST of probabilistic programs for so-called *Prob-solvable* loops, which do not allow for nested or sequential loops or non-determinism. So both [61] and [48] are orthogonal to our work. We discuss [10,50,62] below.

*Implementation* We implemented our analysis in a new version of our tool KoAT [18]. KoAT is an open-source tool written in OCaml, which can also be downloaded as a Docker image and accessed via a web interface [43].

Given a PIP, the analysis proceeds as in Alg. 1. The preprocessing in Line 1 adds invariants to guards (using APRON [39] to generate (non-probabilistic) invariants), unfolds transitions [19], and removes unreachable locations, transitions with probability 0, and transitions with unsatisfiable guards (using Z3 [49]).

**Input:** PIP $(\mathcal{PV}, \mathcal{L}, \mathcal{GT}, \ell_0)$
1 preprocess the PIP
2 $(\mathcal{RB}, \mathcal{SB}) \leftarrow$ perform non-probabilistic analysis using [18]
3 $(\mathcal{RB}_{\mathbb{E}}, \mathcal{SB}_{\mathbb{E}}) \leftarrow$ lift $(\mathcal{RB}, \mathcal{SB})$ to an expected bound pair with Thm. 10
4 **repeat**
5     **for all** SCCs $C$ of the general result variable graph in topological order **do**
6         **if** $C = \{\alpha\}$ is trivial **then** $\mathcal{SB}'_{\mathbb{E}} \leftarrow$ improve $\mathcal{SB}_{\mathbb{E}}$ for $C$ by Thm. 23
7         **else** $\mathcal{SB}'_{\mathbb{E}} \leftarrow$ improve $\mathcal{SB}_{\mathbb{E}}$ for $C$ by Thm. 25
8         **for all** $\alpha \in C$ **do** $\mathcal{SB}_{\mathbb{E}}(\alpha) \leftarrow \min\{\mathcal{SB}_{\mathbb{E}}(\alpha), \mathcal{SB}'_{\mathbb{E}}(\alpha)\}$
9     **for all** general transitions $g \in \mathcal{GT}$ **do**
10         $\mathcal{RB}'_{\mathbb{E}} \leftarrow$ improve $\mathcal{RB}_{\mathbb{E}}$ for $\mathcal{GT}_{>} = \{g\}$ by Thm. 16
11         $\mathcal{RB}_{\mathbb{E}}(g) \leftarrow \min\{\mathcal{RB}_{\mathbb{E}}(g), \mathcal{RB}'_{\mathbb{E}}(g)\}$
12 **until** no bound is improved anymore
**Output:** $\sum_{g \in \mathcal{GT}} \mathcal{RB}_{\mathbb{E}}(g)$

**Algorithm 1:** Overall approach to infer bounds on expected runtimes

We start by a non-probabilistic analysis and lift the resulting bounds to an initial expected bound pair (Lines 2 and 3). Afterwards, we first try to improve the expected size bounds using Thm. 23 and 25, and then we attempt to improve the expected runtime bounds using Thm. 16 (if we find a PLRF using Z3). To determine the "minimum" of the previous and the new bound, we use a heuristic which compares polynomial bounds by their degree. While we over-approximated the maximum of expressions by their sum to ease readability in this paper, KoAT also uses bounds containing "min" and "max" to increase precision.

This alternating modular computation of expected size and runtime bounds is repeated so that one can benefit from improved expected runtime bounds when computing expected size bounds and vice versa. We abort this improvement of expected bounds in Alg. 1 if they are all finite (or when reaching a timeout).

To assess the power of our approach, we performed an experimental evaluation of our implementation in KoAT. We did not compare with the tool of [62], since [62] expects the program to be annotated with already computed invariants. But for many of the examples in our experiments, the invariant generation tool [56] used by [62] did not find invariants strong enough to enable a meaningful analysis (and we could not apply APRON [39] due to the different semantics of invariants).

Instead, we compare KoAT with the tools Absynth [50] and eco-imp [10] which are both based on a conceptionally different *backward-reasoning* approach. We ran the tools on all 39 examples from Absynth's evaluation in [50] (except `recursive`, which contains non-tail-recursion and thus cannot be encoded as a PIP), and on the 8 additional examples from the artifact of [50]. Moreover, our collection has 29 additional benchmarks: 14 examples that illustrate different aspects of PIPs, 5 PIPs based on examples from [50] where we removed assumptions, and 10 PIPs based on benchmarks from the *TPDB* [59] where some transitions were enriched with probabilistic behavior. The *TPDB* is a collection of typical programs used in the annual *Termination and Complexity Competition* [31]. We ran the experiments on an iMac with an Intel i5-2500S CPU and 12 GB of RAM under macOS Sierra for Absynth and NixOS 20.03 for KoAT and eco-imp. A timeout of 5 minutes per

| **Bound** | KoAT | Absynth | eco-imp |
|---|---|---|---|
| $\mathcal{O}(1)$ | 6 | 6 | 6 |
| $\mathcal{O}(n)$ | 32 | 32 | 29 |
| $\mathcal{O}(n^2)$ | 3 | 8 | 9 |
| $\mathcal{O}(n^{>2})$ | 0 | 0 | 0 |
| EXP | 0 | 0 | 0 |
| $\infty$ | 5 | 0 | 2 |
| TO | 0 | 0 | 0 |

Fig. 2: Results on benchmarks from [50]

| **Bound** | KoAT | Absynth | eco-imp |
|---|---|---|---|
| $\mathcal{O}(1)$ | 2 | 1 | 2 |
| $\mathcal{O}(n)$ | 10 | 3 | 6 |
| $\mathcal{O}(n^2)$ | 12 | 1 | 6 |
| $\mathcal{O}(n^{>2})$ | 2 | 0 | 0 |
| EXP | 1 | 0 | 0 |
| $\infty$ | 2 | 15 | 12 |
| TO | 0 | 9 | 3 |

Fig. 3: Results on our new benchmarks example was applied for all tools. The average runtime of successful runs was 4.26 s for KoAT, 3.53 s for Absynth, and just 0.93 s for eco-imp.

Fig. 2 and 3 show the generated asymptotic bounds, where $n$ is the maximal absolute value of the program variables at the program start. Here, "$\infty$" indicates that no finite time bound could be computed and "TO" means "timeout". The detailed asymptotic results of all tools on all examples can be found in [43, 47].

Absynth and eco-imp slightly outperform KoAT on the examples from Absynth's collection, while KoAT is considerably stronger than both tools on the additional benchmarks. In particular, Absynth and eco-imp outperform our approach on examples with nested probabilistic loops. While our modular approach can analyze inner loops separately when searching for probabilistic ranking functions, Thm. 16 then requires *non-probabilistic* time bounds for all transitions entering the inner loop. But these bounds may be infinite if the outer loop has probabilistic behavior itself. Moreover, in contrast to our work and [10], the approach of [50] does not require weakly monotonic bounds.

On the other hand, KoAT is superior to Absynth and eco-imp on large examples with many loops, where only a few transitions have probabilistic behavior (this might correspond to the typical application of randomization in practical programming). Here, we benefit from the modularity of our approach which treats loops independently and combines their bounds afterwards. Absynth and eco-imp also fail for our leading example of Fig. 1, while KoAT infers a quadratic bound. Hence, the tools have particular strengths on orthogonal kinds of examples.

KoAT's source code is available at https://github.com/aprove-developers/KoAT2-Releases/tree/probabilistic. To obtain a KoAT artifact, see https://aprove-developers.github.io/ExpectedUpperBounds/ for a static binary and Docker image. This web site also provides all examples from our evaluation, detailed outputs of our experiments, and a *web interface* to run KoAT directly online.

*Conclusion* We presented a new modular approach to infer upper bounds on the expected runtimes of probabilistic integer programs. To this end, non-probabilistic and expected runtime and size bounds on parts of the program are computed in an alternating fashion and then combined to an overall expected runtime bound. In the evaluation, our tool KoAT succeeded on 91% of all examples, while the main other related tools (Absynth and eco-imp) only inferred finite bounds for 68% resp. 77% of the examples. In future work, it would be interesting to consider a modular combination of these tools (resp. of their underlying approaches).

*Acknowledgements* We thank Carsten Fuhs for discussions on initial ideas.
